# Antiamnesic Effects of a Hydroethanolic Extract of *Crinum macowanii* on Scopolamine-Induced Memory Impairment in Mice

**DOI:** 10.1155/2015/242505

**Published:** 2015-10-08

**Authors:** Andrew T. Mugwagwa, Louis L. Gadaga, William Pote, Dexter Tagwireyi

**Affiliations:** ^1^Drug and Toxicology Information Service (DaTIS), School of Pharmacy and Department of Clinical Pharmacology, College of Health Sciences, University of Zimbabwe, P.O. Box A 178, Avondale, Harare, Zimbabwe; ^2^Department of Preclinical Veterinary Studies, Faculty of Veterinary Sciences, University of Zimbabwe, P.O. Box MP, Mount Pleasant, Harare, Zimbabwe

## Abstract

*Crinum macowanii* has been found to contain alkaloids that have activity against acetylcholinesterase enzyme *in vitro*. The present study was undertaken to investigate the *in vivo* ability of hydroethanolic crude extract of *Crinum macowanii* to ameliorate memory impairment induced by scopolamine. Thirty-six male Balb/c mice weighing around 25–35 g were employed in the present investigation. Y-maze and novel object recognition apparatus served as the exteroceptive behavioural models, and scopolamine-induced amnesia served as the interoceptive behavioural model. *C. macowanii* (10, 20, and 40 mg/kg p.o.) was administered in single doses to the mice. Donepezil (3 mg/kg p.o.) was used as a positive control agent. *C. macowanii* extract reversed the amnesia induced by scopolamine as indicated by a dose-dependent increase in spontaneous alternation performance in the Y-maze task. *C. macowanii* 40 mg/kg showed significant activity (*p* < 0.05 versus negative control), comparable to that of the positive control. *C. macowanii* also showed memory-enhancing activity against scopolamine-induced memory deficits in the long-term memory novel object recognition performance as indicated by a dose-dependent increase in the discrimination index. The results indicate that the hydroethanolic extract of *C. macowanii* may be a useful memory restorative mediator in the treatment of cognitive disorders such as Alzheimer's disease.

## 1. Introduction

Dementia is a general term for the loss of memory and other intellectual abilities serious enough to disrupt daily activities later in life [1]. Common types of dementia include Alzheimer's disease (AD), cerebrovascular dementia, dementia with Lewy bodies, frontotemporal dementia, and Parkinson's disease [2]. AD is the most common type of dementia and is responsible for 50–60% of all cases [3]. AD is a progressive neurodegenerative disease of the brain characterized by memory loss, behavioural changes, and associative signs and symptoms [4]. Memory loss is usually the initial symptom of the disease.

The most accepted therapeutic approach in AD has been the use of acetylcholinesterase inhibitors (AChEIs). This blocks the activity of an enzyme called acetylcholinesterase which normally breaks down acetylcholine (ACh). Inhibition of this enzyme prevents the breakdown of ACh that is known to be lacking in the AD brain. Although effective in providing symptomatic treatment, these drugs are nonselective and thus may lead to an overstimulation of cholinergic systems outside of the brain and cause a variety of cholinergic manifestations [5]. Selective AChEIs, free of dose-limiting side effects, are until now not available. Taking into account these therapeutic limitations of AD, there is a compelling need to discover and develop new and better drugs for this devastating disorder.

Medicinal plants have a long history in the treatment of various illnesses due to their therapeutic value. Since the allopathic approach to medicine is yet to provide a radical cure for AD [3], the world is tending to explore the utility of medicinal plants in the treatment of this disorder. Plants of the Amaryllidaceae family have been shown to contain alkaloids with pharmacological activities such as anticholinergic, analgesic, antitumour, and antiviral [6, 7]. This development has prompted us to investigate the memory-enhancing activity of plant species from this family. One of the plants that has been investigated by our group is* Crinum macowanii* (*C*.* macowanii*) which is referred to as* dururu* by Shona or* umduze* by Ndebele communities. In Zimbabwe,* C*.* macowanii* has been used to treat backaches, emesis, and venereal disease [8]. Alkaloids of* C*.* macowanii* have been screened for acetylcholinesterase inhibition activity* in vitro* and the alkaloid 1-*O*-acetyllycorine was found to be twice as potent as galanthamine [9]. Therefore, the aim of the present study was to investigate the possible memory-enhancing properties of hydroethanolic crude extract of* C*.* macowanii* in scopolamine-induced amnesiac mice.

## 2. Materials and Methods

### 2.1. Plant Collection

*C*.* macowanii* was collected in December 2012 at the University of Zimbabwe (UZ) grounds, Harare. The plant sample (whole plant with bulb, leaves, and flower) was authenticated by a taxonomist from the Botanical Gardens and National Herbarium in Harare, and a voucher specimen was kept in Pharmaceutical Chemistry Laboratory, School of Pharmacy. A total number of 18 bulbs were collected for this study.

### 2.2. Preparation of Crude Extract

The fresh bulbs (18 bulbs) were washed and peeled to facilitate drying and then shade-dried for 3 weeks. The partially air dried bulb scales were then oven-dried in an oven (Baird and Tatlock, UK) at 55°C until all the scales were papery dry. The dried scales were ground in a Thomas Scientific Mill Model 4 (Thomas-Wiley Laboratory, USA) (sieve size = 1 mm) until a fine powder (800 g) was obtained. The powder was then macerated (1 : 10 w/v) in aqueous ethanol (70% v/v, up to 8 L). The mixture was left for 72 hours at room temperature with occasional shaking for the extraction of alkaloids to be complete. After 72 hours, the extract was filtered using a mutton cloth to remove the bulky material. The filtrate obtained was further vacuum filtered through Merck number 454 filter paper to remove the finer particulates. After the filtration process, the aqueous-ethanolic extract volume had been reduced from an initial volume of 8 L to approximately 5 L. The 5 L solvent-extract mixture was further reduced in volume by rotary evaporation with a Heidolph instrument, Rotavapor 4000 (Heidolph, Germany), to a thick brown paste (120 mL). The speed of the Rotavapor ranged from 90 to 150 revolutions per minute (rpm) and the temperature was varied from 60 to 90°C. Finally, the 120 mL thick paste was freeze dried from −35 to −15°C using Heto Lab Equipment, Heto Freeze Drier 3 (Heto-Holten A/S, Denmark) for 48 hours. A semisolid crude extract weighing 89.89 g was obtained. The semisolid extract was notably hygroscopic and therefore it was placed in a tightly sealed container and kept in a desiccator. The desiccator was stored in a cool dark place until required for use in behavioural experiments.

### 2.3. Animals and Animal Husbandry

Male Balb/c mice (25–35 g at the beginning of the experiment) were purchased from the animal house at the University of Zimbabwe. The animals were housed in the animal holding room, veterinary department. An adaptation period of at least 7 days was allowed for the mice to get accustomed to the experimenter's handling and also to the animal room conditions prior to the start of the behavioral experiments. The animal room was maintained under standard animal housing conditions (12 : 12 hour light/dark cycle at room temperature, typical Harare summer temperature ≈ 25 ± 5°C (February, 2013)). They had free access to the standard pellet diet (designed and supplied by National Foods, Zimbabwe) and tap water* ad libitum* in their home cages. The mice were housed 7 per cage and bedding was changed at least three times a week. Experiments were carried out during day time between 08:00 and 17:00 hours. Authorization to use laboratory animals in the research project was granted by the Joint Research and Ethical Committee (Approval number JREC/333/12).

### 2.4. Drugs and Reagents

Scopolamine (hyoscine butylbromide) (Pharmacare Limited, SA), donepezil hydrochloride (Actavis Limited, India), and crude extract of* C*.* macowanii* were used. Injection of scopolamine was diluted in normal saline (Adcock Ingram). Donepezil tablets were dissolved in normal saline. The* C*.* macowanii* extract was dissolved in normal saline over a water bath to ensure complete dissolution.

### 2.5. Grouping and Treatment Schedule

Mice were randomly put into six different groups (*n* = 6). The tail of each mouse was marked with a permanent marker for easy identification. Treatment administration in the six groups was follows:

 group 1: normal saline, i.p. and p.o. (vehicle control);
 group 2: scopolamine i.p. and normal saline p.o. (negative control);
 group 3:* Crinum macowanii* 10 mg/kg, p.o. + scopolamine 1 mg/kg, i.p.;
 group 4:* Crinum macowanii* 20 mg/kg, p.o. + scopolamine 1 mg/kg, i.p.;
 group 5:* Crinum macowanii* 40 mg/kg, p.o. + scopolamine 1 mg/kg, i.p.;
 group 6: donepezil 3 mg/kg, p.o. + scopolamine 1 mg/kg, i.p. (positive control).

The volume of oral (p.o.) and intraperitoneal (i.p.) administrations was 1 mL/100 g bw of mice. For each behavioural experiment performed in this study, normal saline p.o.,* C*.* macowanii* (10 mg/kg, 20 mg/kg, and 40 mg/kg p.o.), and donepezil (3 mg/kg p.o.) were administered to mice 1 hour before each trial. Normal saline i.p. and scopolamine (1 mg/kg i.p.) were administered 30 minutes before each trial.

### 2.6. Behavioural Tests

#### 2.6.1. Y-Maze Spontaneous Alternation Behaviour (SAB)

Short-term memory (STM) function was assessed by recording SAB in a Y-maze according to the procedure described by Hughes (2004). The Y-maze consisted of three arms of equal size, labelled as A, B, and C, respectively. Each arm was 19 cm long, 5 cm wide, and 14.5 cm high and was oriented at an angle of 120° from the other two. Mice were placed within one arm, and the sequence (e.g., CBABCC) and the number of arm entries were recorded manually for each mouse over a five-minute session. Alternation was defined as consecutive entries by a mouse into the three different arms. An arm entry was judged to be completed when the hind paws of the mouse were completely placed in an arm. The arena was cleaned using 70% v/v ethanol between trials so as to avoid olfactory cues. The initial arm of the maze was also changed within mice of the same group in order to avoid bias of arm placement. The parameters measured included number of arm entries, same arm returns (SAR), and alternate arm returns (AAR). Percentage of spontaneous alternation performance (%SAP) was determined using the equation

(1) %SAP⁡=Actual alternationstotal alternationsPossible alternationstotal number of arm entries−2×100.

#### 2.6.2. Novel Object Recognition Task

Novel object recognition (NOR) task consisted of habituation, sample, and test phases. The arena (43 cm × 31 cm × 16 cm) was made of plastic. In the sample phase, each mouse was placed in an open field with two identical objects (plastic motorbikes) for five minutes. The mouse was then returned to its home cage. The arena and objects were cleaned with 70% v/v ethanol between trials to avoid olfactory cues. For short-term memory (STM), the test phase was performed five minutes after the sample phase. In the test phase, each mouse was placed again in the open field in which one of the identical objects had been replaced with a novel object (plastic cube). The location of the object was counterbalanced so that one half of the mice in each group saw the novel object on the left side of the box arena, and the other half saw the novel object on the right side of the box arena to eliminate bias of sides. A wash-out period of five days was allowed before the NOR task was used to assess long-term memory in mice. The procedure was the same as that for STM except that mice were presented in the test phase 24 hours after the sample phase exposure. The time spent exploring each object in each phase was recorded manually using a stopwatch. A mouse was scored as exploring when its head was oriented towards the object within a distance of 2 cm or when the nose was in contact with the object. Parameters measured included the time (in seconds) spent exploring familiar object (*Tf*), time (in seconds) spent exploring the novel object (*Tn*), and total time (in seconds) spent exploring both objects (*Tf* + *Tn*). Percentage of discrimination index  (%DI) was determined using the following equation:

(2) $$\begin{array}{c} \text{DI}\left(\;\%\;\right)=\frac{Tn}{\left(Tn+Tf\right)}\times 100\%. \end{array}$$

#### 2.6.3. Data Analysis

Data obtained from* in vivo* experiments were expressed as the mean ± standard error of mean (SEM) and plotted with GraphPad Prism software (version 5.03) for graphical representation. Statistical differences between the treatments and control groups were evaluated using one-way analysis of variance (ANOVA) followed by Dunnett's Multiple Comparison tests in GraphPad Prism (version 5.03). Two-way repeated measures (mixed model) ANOVA followed by Bonferroni posttests was used to compare the two objects in the object recognition task in GraphPad Prism (version 5.03). Differences were considered significant if *p* < 0.05 [^*∗*^ *p* < 0.05; ^*∗∗*^ *p* < 0.01; ^*∗∗∗*^ *p* < 0.001].

## 3. Results

### 3.1. Y-Maze Performance

**Figure 1:**
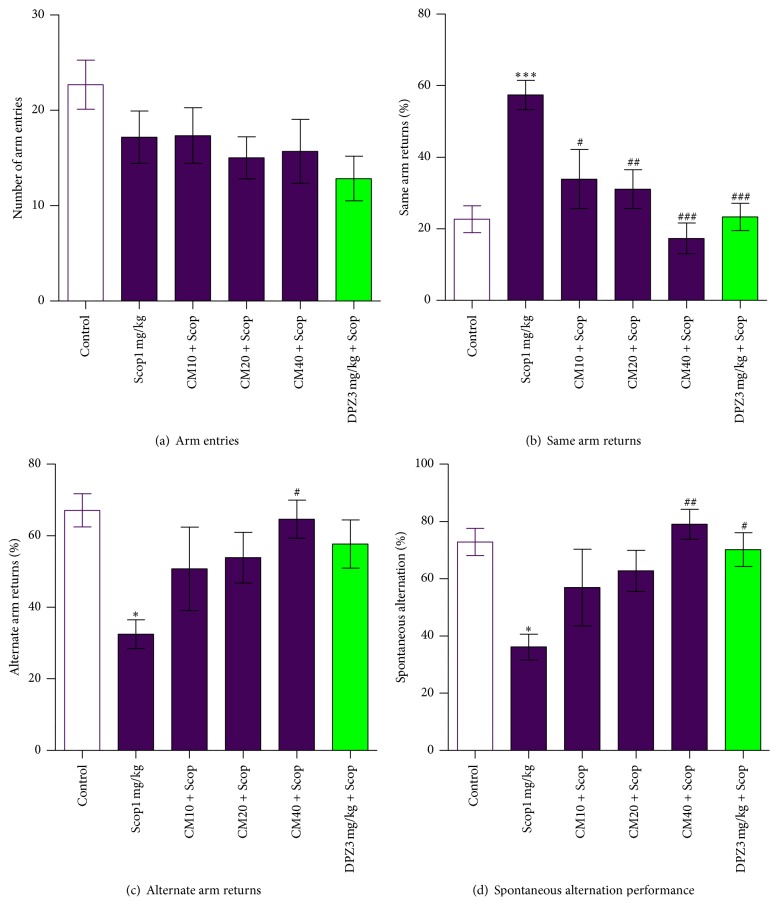
Effect of* Crinum macowanii* (CM) (10, 20, and 40 mg/kg) on the Y-maze task: (a) total number of arm entries, (b) same arm returns, (c) alternate arm returns, and (d) spontaneous alternation performance. Data represent mean ± SEM, *n* = 6. ^**∗**^ *p* < 0.05 versus control and ^#^ *p* < 0.05 versus Scop1 mg/kg group and ^##^ *p* < 0.01 versus Scop1 mg/kg group (one-way analysis of variance followed by Dunnett's Multiple Comparison tests).

The results obtained with the Y-maze task are shown in Figure 1. The number of arm entries (NAEs) was observed in the negative control group (Figure 1(a)). However, the total NAEs were not significantly different among all groups (*p* > 0.05). There was a dose-dependent decrease in the percentage of same arm returns (%SAR) among* Crinum macowanii* (CM) treated groups (Figure 1(b)). Notable differences were observed among the CM treated groups relative to the Scop1 mg/kg group (negative control). CM40 mg/kg group had the lowest %SAR which was more or less the same as that of the DPZ3 mg/kg + Scop group (positive control). There was an expected dose-dependent increase in the alternate arm returns percentage (%AAR) among the* Crinum macowanii* (CM) treated groups (Figure 1(c)). The %AAR for CM40 mg/kg group was high and comparable to that for the positive control group. A significant difference was observed between the CM40 mg/kg and Scop1 mg/kg (*p* < 0.05). Scop1 mg/kg group was the lowest %AAR and a significant difference was revealed when it was compared with the vehicle control group (*p* < 0.05). There was a dose-dependent increase in the percentage of spontaneous alternation performance (%SAP) among* Crinum macowanii* (CM) treated groups (Figure 1(d)). %SAP for the CM40 mg/kg group was high, comparable to that of the positive control group (DPZ3 mg/kg + Scop). A significant difference was observed between the CM40 mg/kg + Scop and Scop1 mg/kg groups (*p* < 0.01). %SAP between the Scop1 mg/kg and the vehicle and positive controls group had significant differences (*p* < 0.05).

### 3.2. Novel Object Recognition Performance

**Figure 2:**
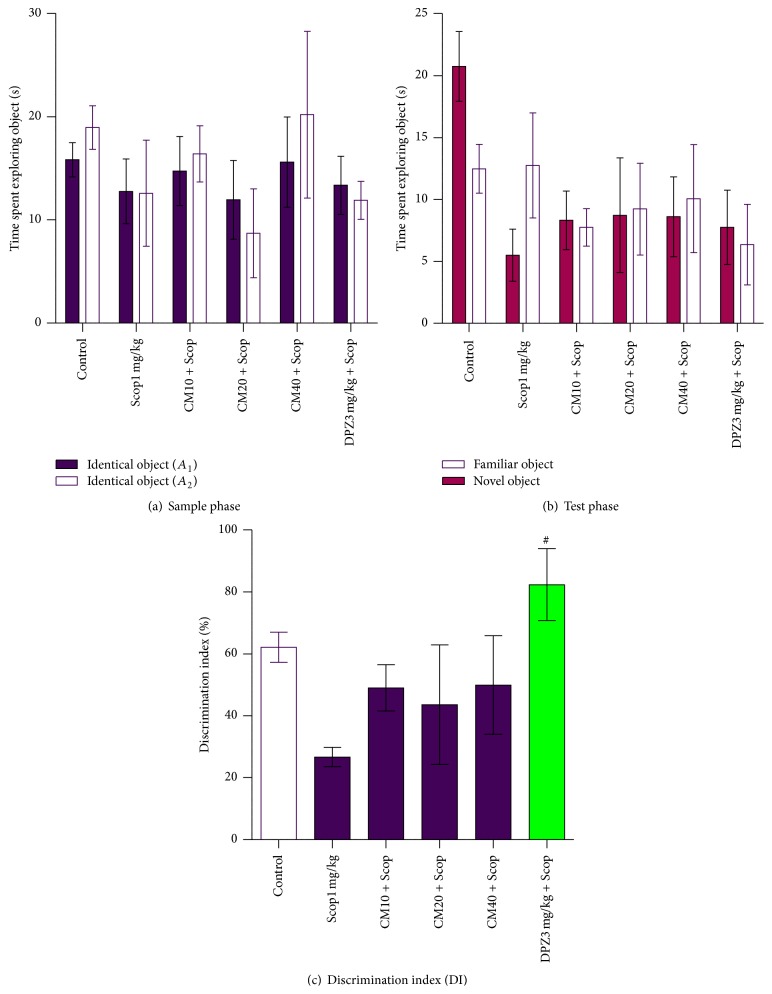
Effect of* Crinum macowanii *(CM) (10, 20, and 40 mg/kg) on the short-term memory object recognition task: (a) exploration times in the sample phase, (b) exploration times in the test phase, and (c) discrimination index. ^#^ *p* < 0.05 versus Scop1 mg/kg group. ((a) and (b)) Two-way repeated measures (mixed model) ANOVA followed by Bonferroni posttests. (c) One-way analysis of variance followed by Dunnett's Multiple Comparison tests.

**Figure 3:**
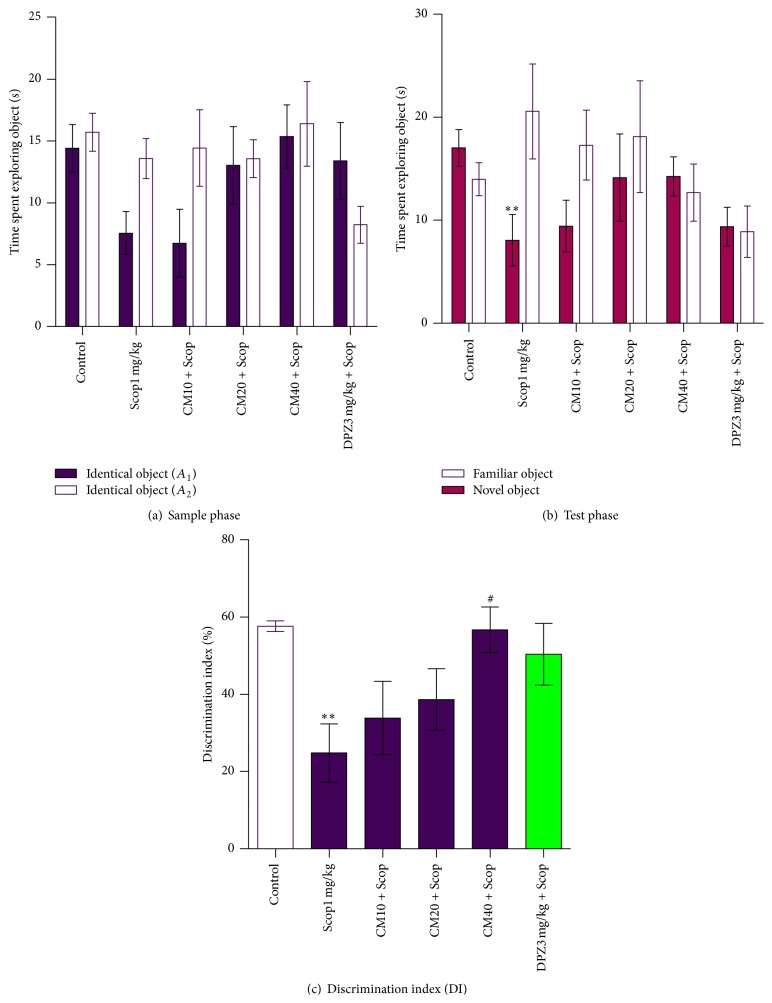
Effect of* Crinum macowanii* (CM) (10, 20, and 40 mg/kg) on the long-term memory object recognition task: (a) exploration times in the sample phase, (b) exploration times in the test phase, and (c) discrimination index. ^#^ *p* < 0.05 versus Scop1 mg/kg group; ^*∗∗*^ *p* < 0.01 versus control group. ((a) and (b)) Two-way repeated measures (mixed model) ANOVA followed by Bonferroni posttests. (c) One-way analysis of variance followed by Dunnett's Multiple Comparison tests.

The results obtained with the Y-maze task are shown in Figures 2 and 3. In the sample phase of the short-term memory task, none of the animal-treated groups showed statistical difference in the total time spent exploring two objects (Figure 2(a)). Neither was there a statistical difference in the time spent exploring each identical object among the animal-treated groups (*p* > 0.05).

In the test phase, the negative control, CM10 mg/kg, and the DPZ3 mg/kg + Scop groups spent a longer time exploring the novel object than exploring the familiar one although the difference was not statistically significant (Figure 2(b)). A preference for the novel object was more or less the same among the* Crinum macowanii* (CM) treated groups. The Scop1 mg/kg group spent a notable longer time exploring the familiar phase than exploring the novel one although the difference was not statistically significant (*p* > 0.05).

There was no increase in the percentage discrimination index (%DI) among the test groups (Figure 2(c)). The percentage discrimination index for the Scop1 mg/kg, CM10 mg/kg, CM20 mg/kg, and CM40 mg/kg groups was below 50%. The %DI for the positive control (DPZ3 mg/kg + Scop) group was notably higher. One-way analysis of variance (ANOVA) followed by Dunnett's Multiple Comparison tests revealed a significant difference between the Scop1 mg/kg and the DPZ3 mg/kg + Scop groups (*p* < 0.05).

Similar to the sample phase in the short-term memory task, none of the animal-treated groups showed a significant difference in the total time spent exploring two objects in the long-term memory task (Figure 3(a)). Neither was there a significant difference in the time spent exploring each identical object among the animal-treated groups (*p* > 0.05).

A slight dose-dependent increase in the preferences to the novel object was observed among the* Crinum macowanii* (CM) treated groups. The CM40 mg/kg groups spent a longer time exploring the novel object than exploring the familiar one (Figure 3(b)). Although the difference was not significant, the preference to the novel object in the CM40 mg/kg group was comparable to that of the positive control group (DPZ3 mg/kg + Scop). Two-way repeated measures (mixed model) ANOVA followed by Bonferroni posttests revealed significant differences between the exploration times of the novel and familiar objects in the Scop1 mg/kg group (*p* < 0.01). A dose-dependent increase in the discrimination index percentage (%DI) was observed among the* Crinum macowanii* (CM) treated groups. The %DI for the CM40 mg/kg group was high, above that of the DPZ3 mg/kg + Scop group (Figure 3(c)). A significant difference in the %DI was observed between the CM40 mg/kg and Scop1 mg/kg groups (*p* < 0.05). %DI for the Scop1 mg/kg group was notably lower than all other groups and there was a significant difference when it was compared with the vehicle control.

## 4. Discussion

The present study investigated the memory-enhancing activity of hydroethanolic crude extract of* Crinum macowanii* bulbs in mice models. The anticholinesterase inhibitory activity of some of its alkaloids such as 1-*O*-acetyllycorine, lycorine, and crinine [6, 9] prompted us to carry out this investigation. Progressive neurodegeneration in the brain, leading to the depletion of acetylcholine (ACh) stores is the primary cause of memory loss in the ageing population. Drugs or plants with acetylcholinesterase (AChE) activity can thus increase levels of ACh that are known to be depleted in neurodegenerative disorders such as Alzheimer's disease (AD) by inhibiting AChE, an enzyme that degrades ACh [10].

In humans, memory is generally accessed through spoken or written languages, whereas, in laboratory animals, cognitive functions must be accessed through behavioral models [11]. Two such models are the spontaneous alternation performance (SAP) in the Y-maze and object recognition performance. The SAP and object recognition tasks used in this study have effects on the hippocampus region of the brain which is also associated with neurological damage [12, 13]. Moreover, the scopolamine used in this study to induce amnesia has a high selectivity for the muscarinic receptors, especially the M_1_ receptor subtypes found in the hippocampus [14].

Spontaneous alternation is a measure of exploration behaviour in mice and is a reliable screening model to test effects of drugs including natural products against scopolamine-induced memory impairments [15]. A mouse with an impaired short-term memory (STM) cannot recall which arm it has just visited and thus tends to exhibit decreased spontaneous alternation [16]. In the present study, we measured spontaneous alternation behaviour in the Y-maze test to appraise working memory.

Using the spontaneous alternation model, administration of scopolamine (1 mg/kg) significantly decreased spontaneous alternation performance demonstrating STM impairment behaviour in mice (Figure 1(d)). The administration of hydroethanolic crude extract of* Crinum macowanii* significantly increased spontaneous alternation performance decreased by scopolamine treatment in the CM40 mg/kg + Scop treated group and this was similar to the effect of the positive control (DPZ3 mg/kg + Scop) group. This result indicates memory-enhancing activity of the* Crinum macowanii* extract in scopolamine-induced memory deficits.

Donepezil, an antidementia drug, increases acetylcholine (ACh) levels by inhibition of acetylcholinesterase (AChE) [10]. Pretreatment with donepezil prevented the inhibitory effect of scopolamine. Since the spontaneous alternation performance of the CM40 mg/kg + Scop group was comparable to that of the donepezil pretreated group, it may be postulated that extract of* Crinum macowanii* at 40 mg/kg dose has similar mechanism of action to donepezil. These data concerning oral administration of* Crinum macowanii* in mice agree with reports that* Crinum macowanii* contains alkaloids that may attenuate memory loss symptoms by inhibiting the activity of AChE and hence elevate the levels of ACh in the brain [17]. Moreover, the CM40 mg/kg + Scop group showed greater percentage of spontaneous alternation as compared to the DPZ3 mg/kg + Scop group, which might be due to more selective inhibition of AChE in the hippocampus by alkaloids in* Crinum macowanii* at a dose of 40 mg/kg. Spontaneous alternation performance among* Crinum macowanii* treated groups showed an increasing dose-dependent effect, suggesting that memory-enhancing activity of* Crinum macowanii* on memory impairments induced by scopolamine may be connected to the inhibition of AChE activity. The total number of arm entries, alternate arm returns (AAR), and same arm returns (SAR) parameters was all recorded so as to come up with the percentage of spontaneous alternation performance. The results demonstrated that the total number of arm entries in Y-maze spontaneous alternation performance test was not significantly different among all groups (Figure 1(a)). This might suggest that the changes in spontaneous alternation behavior were not due to locomotor deficits. In general, high SAR percentages indicate memory impairment induced by scopolamine. In this study, a high SAR in the Scop1 mg/kg group indicated the memory dysfunction activity of scopolamine and lower SAR in the CM40 mg/kg + Scop and DPZ3 mg/kg + Scop groups indicated the ameliorative effects of* Crinum macowanii* and donepezil on memory deficit (Figure 1(b)).

The object recognition task is a highly recognised test for both short-term and long-term memories [18]. In this study, this model was used to test the effectiveness of memory-enhancing compounds (hydroethanolic crude extract of* Crinum macowanii*) against memory impairments induced by scopolamine.

The enhancing effect of* Crinum macowanii* on short-term memory deficit was further investigated in the object recognition test. In the sample phase, none of the animal groups showed significant differences in the time spent exploring each identical object (Figure 2(a)). There was also no significant difference in the total time spent exploring two objects between all scopolamine treated groups and the vehicle control indicating no difference in the power to visually recognise objects. This result suggests that dilation of pupils, impairment of lens accommodation, and blurred vision often associated with scopolamine administration [14], were almost insignificant at 1 mg/kg dose of scopolamine.

In contrast, the test phase clearly revealed that the vehicle control group spent more time exploring a novel object, whereas the Scop1 mg/kg group failed to show a novelty preference, indicating that scopolamine causes impairment of object recognition memory in the short-term memory recognition task (Figure 2(b)). Among the* Crinum macowanii* treated groups, there was no statistical difference in time spent exploring the novel object and the familiar object. This result demonstrates impairment of short-term memory among the test group. The positive control group however showed a novelty preference demonstrating intact memory. As shown in Figure 3(c), the inability of the* Crinum macowanii* treated groups to attenuate scopolamine-induced memory deficits of the novel object recognition task and the enhancing effects of donepezil on the deficits were also confirmed by analysing the data as a discrimination index. Therefore, from the failure of* Crinum macowanii* to affect the memory deficits induced by scopolamine in the short-term object recognition task, it might be considered that* Crinum macowanii* has less capacity to affect the mechanism of action responsive to anticholinesterase drug therapy.

The results obtained in this task however conflicted with those earlier found in the spontaneous alternation performance. The reasons for these discrepancies are unclear. Nevertheless, it should be noted that object recognition tasks are based on spontaneous exploratory activity, and as a result they do not leave out the likelihood of individual animals having a preference for a specific object that is independent of the familiarity/novelty of that item [19]. Moreover, objects used in recognition task should be heavy enough so that animals cannot move them, as well as high enough to prevent animals climbing on them during the two phases [11]. In this model, the familiar objects had some moving parts which subsequently led some of the mice to spend more time with the familiar objects. This might have influenced the outcome of experiments in some animal groups.

Similar to the observations made in the sample phase of the short-term memory object recognition task, none of the animal groups showed significant differences in time spent exploring each identical object. Neither was there a significant difference in the total time spent exploring the two objects between the normal saline treated group (vehicle control) and those pretreated with scopolamine. This result indicates the absence of nonbehavioral effects of scopolamine such as blurry vision and this gives each animal group equal chance to recognise objects. In contrast to the effect of* Crinum macowanii* on the short-term memory object recognition task,* Crinum macowanii* (40 mg/kg) administration had some effect on the scopolamine-induced impairment of long-term memory evaluated as the ability to retain memory after 24 hours. The CM40 mg/kg + Scop group showed novelty preference by spending more time on the novel object relative to the familiar object (Figure 3(b)). As shown in Figure 3(c), the ability of* Crinum macowanii* (40 mg/kg) to ameliorate scopolamine-induced memory deficits of the novel object recognition performance was also confirmed by analysing the data as a discrimination index. The percentage discrimination index for the CM40 mg/kg + Scop group was comparable to that of the negative control group indicating the ability of* Crinum macowanii* (40 mg/kg) to reverse memory deficits induced by scopolamine. On the other hand, the discrimination index for the positive control group was notably lower than expected. The unwillingness of the mice in this group to explore irrespective of familiarity/novelty preference is a possible explanation since the group had the least total exploration time in the test phase as shown in Figure 3(b).

It should be emphasized that the effect of* Crinum macowanii* on both short-term and long-term memory deficits in amnesia animals is likely owing to the inhibition of the activity of acetylcholinesterase, an enzyme primarily responsible for acetylcholine degradation. Because donepezil administration significantly lowered the activity of this enzyme in the brains of amnesia-induced mice, it may be proposed that* Crinum macowanii* administration at a dose of 40 mg/kg had the same effect. These findings suggest that the mechanism(s) underlying the action of* Crinum macowanii* in amnesia-induced animals may be similar to that involved in the action of donepezil. The present findings indicate that* Crinum macowanii* may exhibit a therapeutic effect on short- and long-term memory deficits related to Alzheimer's disease by improving dysfunction of central cholinergic systems [13].

## 5. Conclusion

This study was set out to investigate possible memory-enhancing activity of hydroethanolic crude extract of* Crinum macowanii* bulbs using mice models so as to bring to light its pharmacological effects against memory impairment.* Crinum macowanii* crude extract at high doses demonstrated an ability to enhance memory impaired by scopolamine in the spontaneous alternation performance in the Y-maze but not in the short-term novel object recognition task.* Crinum macowanii* extract at 40 mg/kg demonstrated long-term memory-enhancing activity by ameliorating memory deficits induced by scopolamine in the long-term novel recognition test. Its activity was more than that of donepezil.

## Abbreviations

AAR: Alternate arm returns
AChE: Acetylcholinesterase
AChEIs: Acetylcholinesterase Inhibitors
AD: Alzheimer's disease
LTM: Long-term memory
NAEs: Number of arm entries
NOR: Novel object recognition
SAB: Spontaneous alternation behaviour
SAP: Spontaneous alternation performance
SAR: Same arm returns
STM: Short-term memory.
